# A new osteichthyan from the late Silurian of Yunnan, China

**DOI:** 10.1371/journal.pone.0170929

**Published:** 2017-03-08

**Authors:** Brian Choo, Min Zhu, Qingming Qu, Xiaobo Yu, Liantao Jia, Wenjin Zhao

**Affiliations:** 1 Key Laboratory of Vertebrate Evolution and Human Origins of Chinese Academy of Sciences, Institute of Vertebrate Paleontology and Paleoanthropology, Chinese Academy of Sciences, Beijing, China; 2 School of Biological Sciences, Flinders University, South Australia, Australia; 3 College of Earth Science, University of Chinese Academy of Sciences, Beijing, China; 4 Subdepartment of Evolutionary Organismal Biology, Department of Physiology and Developmental Biology, Uppsala University, Uppsala, Sweden; 5 Center for Advanced Research in Environmental Genomics, University of Ottawa, Ottawa, Canada; 6 Department of Biological Sciences, Kean University, Union, New Jersey, United States of America; Institute of Botany, CHINA

## Abstract

Our understanding of early gnathostome evolution has been hampered by a generally scant fossil record beyond the Devonian. Recent discoveries from the late Silurian Xiaoxiang Fauna of Yunnan, China, have yielded significant new information, including the earliest articulated osteichthyan fossils from the Ludlow-aged Kuanti Formation. Here we describe the partial postcranium of a new primitive bony fish from the Kuanti Formation that represents the second known taxon of pre-Devonian osteichthyan revealing articulated remains. The new form, *Sparalepis tingi* gen. et sp. nov., displays similarities with *Guiyu* and *Psarolepis*, including a spine-bearing pectoral girdle and a placoderm-like dermal pelvic girdle, a structure only recently identified in early osteichthyans. The squamation with particularly thick rhombic scales shares an overall morphological similarity to that of *Psarolepis*. However, the anterior flank scales of *Sparalepis* possess an unusual interlocking system of ventral bulges embraced by dorsal concavities on the outer surfaces. A phylogenetic analysis resolves *Sparalepis* within a previously recovered cluster of stem-sarcopterygians including *Guiyu*, *Psarolepis* and *Achoania*. The high diversity of osteichthyans from the Ludlow of Yunnan strongly contrasts with other Silurian vertebrate assemblages, suggesting that the South China block may have been an early center of diversification for early gnathostomes, well before the advent of the Devonian “Age of Fishes”.

## Introduction

Osteichthyans, comprising the Actinopterygii (ray-finned fishes) and Sarcopterygii (lobe-finned fishes and tetrapods), display a remarkable diversity with over 60,000 extant species. However, their origins and early evolution are obscured by the rarity and typically fragmentary nature of pre-Devonian fossil material [1,2,3].

**Fig 1:**
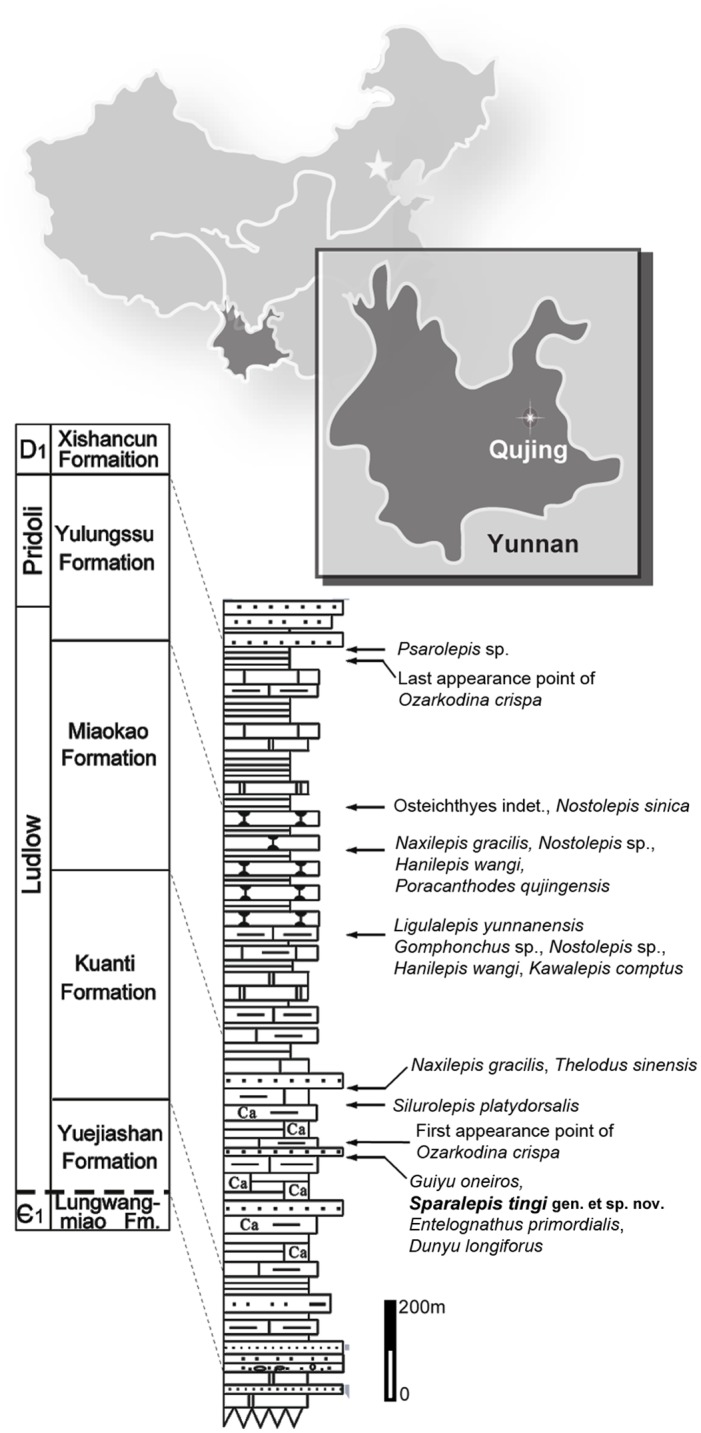
Summary of the Silurian sequence in Qujing (Yunnan, China), displaying the stratigraphic position of *Sparalepis tingi* gen. et sp. nov. and other vertebrate taxa of the Xiaoxiang fauna. Modified after Zhu et al. [8].

Exceptional new data is being revealed via ongoing discoveries from a series of late Silurian (Pridoli and Ludlow) marine strata at Qujing, Yunnan Province, southwestern China. In ascending order, the sequence consists of the Yuejiashan, Kuanti, Miaokao and Yulungssu formations [4,5,6,7]. The fossil assemblage of the lower three formations is collectively referred to as the Xiaoxiang Fauna, in reference to the nearby Xiaoxiang reservoir [5,6]. Of particular importance are collections from the Kuanti Formation of late Ludlow age (Fig 1), revealing a diverse assemblage of early fishes, including the only known articulated fossils of pre-Devonian osteichthyans [8]. A rich fossil invertebrate fauna, including corals, molluscs and trilobites, indicates a productive marine environment [5,7]. Kuanti osteichthyians include *Guiyu oneiros* [8], the large but fragmentary *Megamastax amblyodus* [9], and the scale-based *Naxilepis gracilis* [10]. Other jawed fishes include undescribed acanthodians [5,6] along with various placoderms [10], including *Silurolepis* [11] and the two maxillate placoderms *Entelognathus* [12] and *Qilinyu* [13]. Prior to the discovery of the superbly preserved holotypes of *Guiyu* and *Entelognathus*, pre-Devonian gnathostomes were represented only by highly fragmentary material [1,2,10,14].

The anatomy of these newly discovered Silurian fishes has narrowed the morphological gap between basal sarcopterygians and actinopterygians [8], as well as between early osteichthyans and placoderms [8,12,13]. The unambiguous presence of dermal pelvic girdles in *Guiyu* facilitated the identification of isolated plate-like bones from the Early Devonian Xitun Formation, Yunnan, as the pelvic girdles of the enigmatic osteichthyan *Psarolepis romeri* [15]. Prior to these discoveries, pelvic girdles with extensive dermal components were thought to be restricted to extinct gnathostome groups, specifically the pelvic spines of acanthodians and the fully developed girdles of placoderms [15,16].

The phylogenetic positions of *Guiyu*, *Psarolepis* and other Silurian-Early Devonian osteichthyans are currently under scrutiny. *Guiyu* and *Psarolepis* are usually resolved as stem sarcopterygians [8,15,17,18,19,20,21]. Recent analyses incorporating these taxa plus *Achoania*, another Xitun form [20], resolve a cluster of these three genera as the sister group to the remaining sarcopterygians [8,12]. However these fish manifest combinations of features found in sarcopterygians, actinopterygians (cheek and opercular-gular bone configuration, scale articulation) and non-osteichthyans (median dorsal plates, morphology of appendicular girdles, absence of tooth enamel). Such incongruent character distribution has led to suggestions of an alternative phylogenetic position for these forms as stem-group osteichthyans [15,20,21,22].

**Fig 2:**
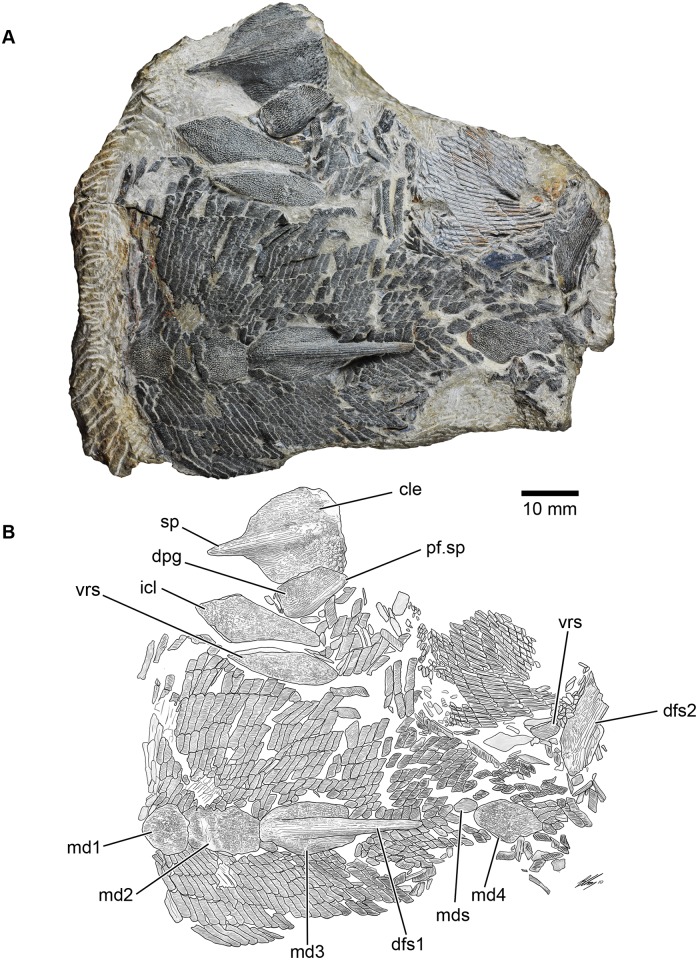
*Sparalepis tingi* gen. et sp. nov., holotype V17915. **A.** Photograph of fossil specimen. **B.** Interpretative diagram of holotype. **Abbreviations: cle**, cleithrum; **dfs1**, 1^st^ dorsal fin spine; **dfs2**, 2^nd^ dorsal fin spine; **dpg**, dermal pelvic girdle; **icl**, interclavicle; **mds** median dorsal scale/scute; **md1-4**, 1^st^-4^th^ median dorsal plates; **pf.sp**, pelvic fin spine; **sp**, pectoral fin spine; **vrs**, ventral ridge scale/scute.

A well preserved fossil osteichthyan, consisting of a partial postcranium (Fig 2), was collected from the Kuanti Formation on the outskirts of Qujing in 2009. It is distinct from other early osteichthyans due to its unusual scale morphology and dermal ornamentation, although it shares several features with *Guiyu* and *Psarolepis*, including spine-bearing dermal pelvic and pectoral girdles, characters also present in placoderms [15]. This new form represents the second known Silurian bony fish with an unambiguously associated dermal pelvic girdle, and its new character combination adds significantly to our understanding of early osteichthyan anatomy and diversity.

## Results

### Systematic paleontology

Osteichthyes (Huxley, 1880)

*Sparalepis tingi* gen. et sp. nov.

rn:lsid:zoobank.org:act:5D97319E-13C8-4702-9785-79C44416FD83

Figs 2, 3, 4, 5, 6, 7, 8 and 9

**Fig 3:**
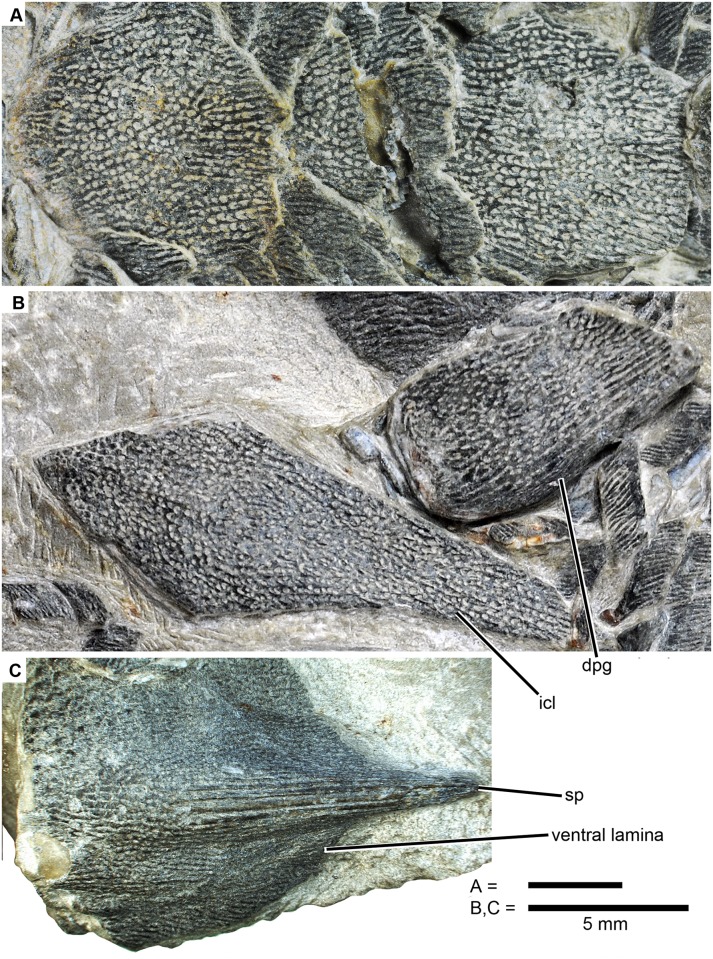
*Sparalepis tingi* gen. et sp. nov., holotype V17915. Detailed images of median dorsal plates and appendicular skeleton. **A.** 1^st^ (left) and 2^nd^ (right) median dorsal plates in dorsal view. **B.** interclavicle and right dermal pelvic girdle. **C.** left cleithrum in flattened ventrolateral view. **Abbreviations: dpg**, dermal pelvic girdle; **icl**, interclavicle; **sp**, pectoral fin spine.

**Fig 4:**
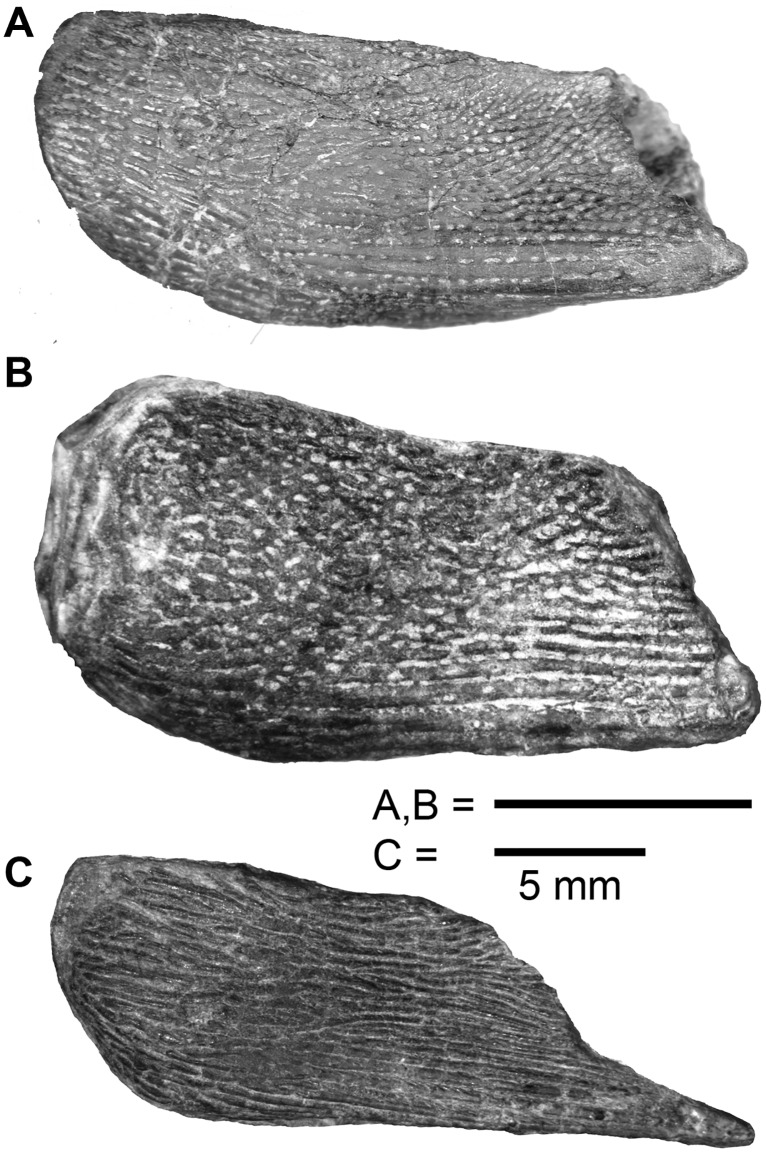
Comparison of dermal pelvic girdles of stem-sarcopterygians. **A.** *Psarolepis romeri* Yu, 1998, from the Lower Devonian Xitun Formation (Lockhovian) of Quijing, Yunnan. V17913.4 (reversed). **B.** *Sparalepis tingi* gen. et. sp. nov., holotype. **C.** *Guiyu oneiros* Zhu et al., 2009, from the Silurian Kuanti Formation (Pridoli) of Quijing, Yunnan. V17914.

**Fig 5:**
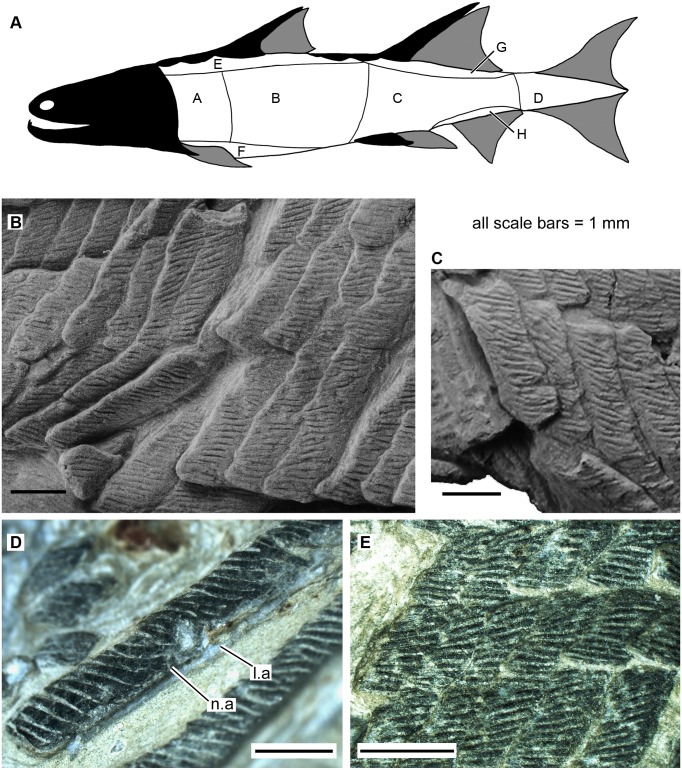
Scales of *Sparalepis tingi* gen. et sp. nov., holotype V17915. **A.** generalised reconstructed silhouette of *Sparalepis* showing scale zones based on the scheme of Esin [30]. **B.** Area A scales from the right flank. **C.** Area A scales from the left flank. **D.** Area B scale in anterolateral view. **E.** Area E scales. **Abbreviations: l.a**, anterior ledge; **n.a**, anterior notch.

**Fig 6:**
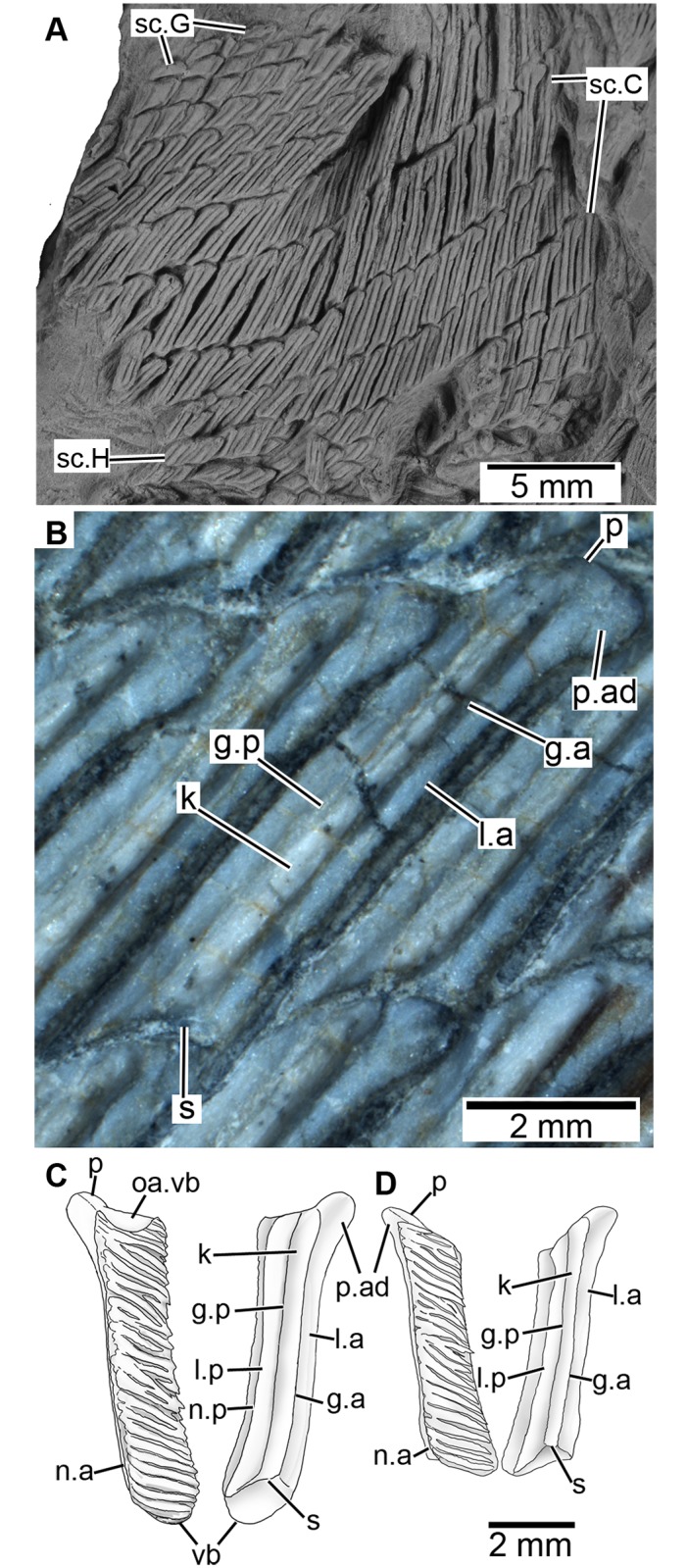
Scales of *Sparalepis tingi* gen. et sp. nov., holotype V17915. **A.** scales from areas C, G and H in basal view. **B.** Basal view of area C scale. Reconstructions of scales from **(C)** area A and **(D)** from the posterior section of area B. **Abbreviations: g.a**, anterior groove; **g.p**, posterior groove; **k**, keel; **l.a**, anterior ledge; **l.p**, posterior ledge; **n.a**, anterior margin of neck; **n.p**, posterior margin of neck; **oa.vb**, overlap area for ventral bulge; **p**, peg; **p.ad**, anterodorsal process; **s**, socket; **vb**, ventral bulge.

**Fig 7:**
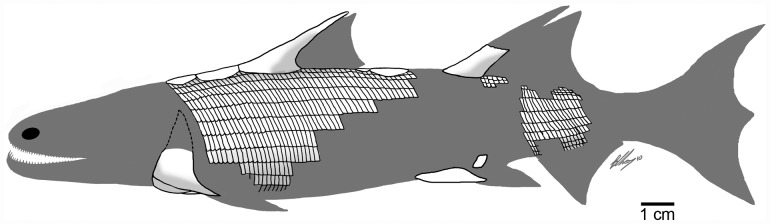
Interpretative reconstruction of *Sparalepis tingi* gen. et sp. nov. Consists of the available fossil elements visible on the holotype superimposed over a generalised silhouette of an early sarcopterygian.

**Fig 8:**
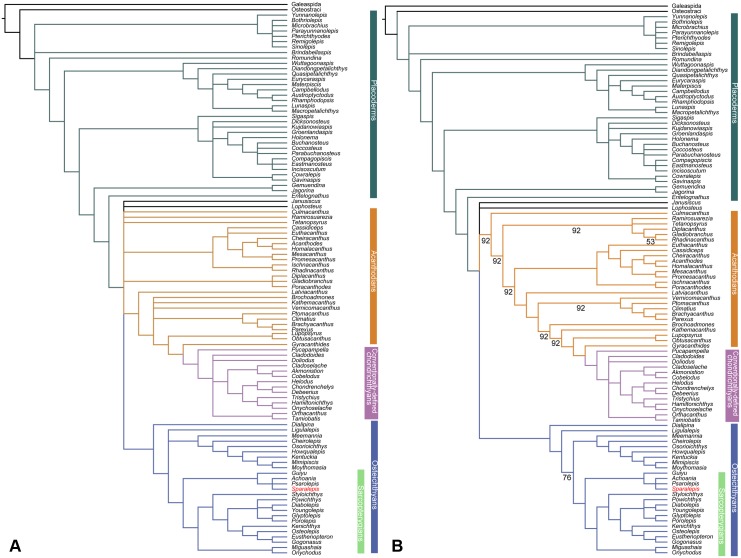
Phylogenetic relationships of *Sparalepis tingi* gen. et sp. nov. Strict consensus (A) and 50% Majority-rule consensus (B) of the 2496 most parsimonious trees recovered in this study. See S1 Fig for synapomorphies at each node.

**Fig 9:**
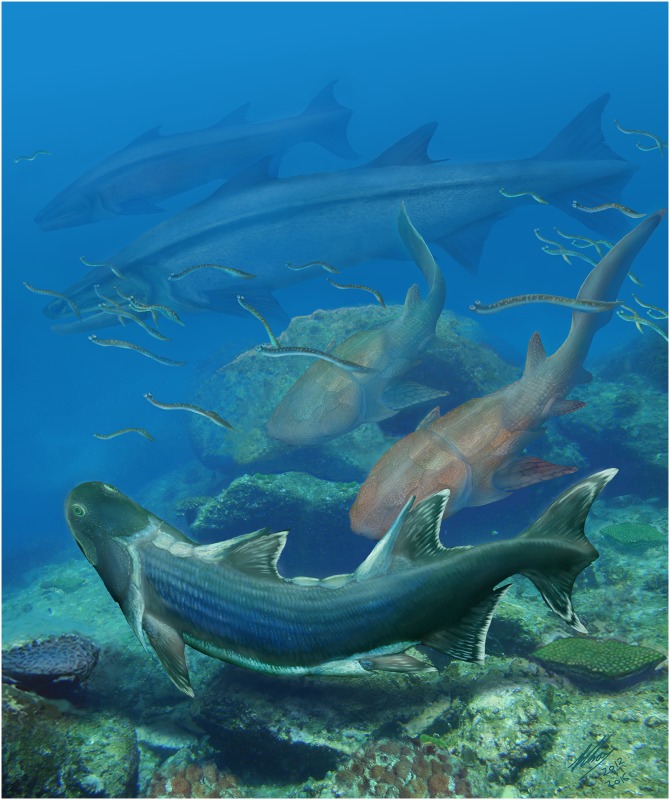
Life restoration of *Sparalepis tingi* (foreground) and other fauna from the Kuanti Formation. Also in the scene are numerous conodont animals, a pair of the maxillate placoderm *Entelognathus* (middle distance) and two examples of the osteichthyan *Megamastax* (background), the largest known Silurian vertebrate. Illustration by Brian Choo, released under Creative Commons Attribution Licence CC BY 4.0, 2016.

#### Holotype and only specimen

V17915, a partial postcranium (Fig 2), with associated cleithrum, interclavicle and pelvic girdle. Institute of Vertebrate Paleontology and Paleoanthropology (IVPP), Beijing.

#### Diagnosis

Bony fish with spine-bearing dermal pectoral and pelvic girdles. Large median dorsal plates, with those immediately anterior to each the two dorsal fins bearing a large spine. Dermal surfaces of the scales and bony plates composed of glossy enamel ornamented with coarse sub-parallel ridges. Large surface pore openings within inter-ridge furrows on the appendicular girdles, gulars and median dorsal plates, but absent on the scales. Thick rhombic scales with a distinct neck separating the crown and the base. Anterior flank scales with a dermal interlocking mechanism of ventral bulges embraced by dorsal concavities on the ventrally adjacent scales. About 30 scale columns in front of the first dorsal fin base.

#### Etymology

Generic name from the Persian *spara* (shield) and the ancient Greek *lepis* (scale), in reference to the resemblance of the scales of the fish to depictions of rectangular wicker shields carried by the Achaemenid *Sparabara* infantry. Specific name after V. K. Ting (1887–1936) for his pioneering work on the geology of Yunnan [4].

**Notes:** The presence of spine-bearing dermal pectoral and pelvic girdles separates *Sparalepis* from all other known osteichthyans except for *Guiyu* and *Psarolepis* [15]. The combination of prominent linear ridges and pore openings on the dermal surfaces of all the larger bones and ridge scutes distinguishes *Sparalepis* from *Guiyu* which possesses ridges only. The scale ornament, consisting of linear ridges and devoid of pores, is similar to that of *Guiyu* [8] and many early actinopterygians [23,24,25,26], but distinct from the porous cosmine-like surface on the scales of *Psarolepis* [27]. The scales of *Sparalepis* are smaller than those of *Guiyu*, with about 30 scale rows anterior to the first dorsal fin against 15 in *Guiyu*. As with *Psarolepis*, the flank scales lack extensive depressed fields and possess necks which separate the crowns from the bases [27]. The ventral bulges and dorsal concavities on the outer surfaces of the anterior flank scales of *Sparalepis* form a unique interlocking system among early osteichthyans.

### Description

The holotype comprises an articulated partial postcranium in flattened dorsal view, from near the base of the skull to about 2 cm beyond the base of the 2^nd^ dorsal fin spine, an interclavicle with an associated ventral scute, a left cleithrum and a right dermal pelvic girdle (Figs 2 and 3). Assuming limited displacement of the posterior section of scales, which is folded perpendicular to the rest of the specimen, the preserved section accounts for a body length of roughly 11cm, suggesting a complete fish of a little over 20 cm.

#### Paired-fin girdles

A left cleithrum (cle) and right dermal pelvic girdle (dpg), both displaced from their original positions, lie adjacent to one another. No endoskeletal structures are visible. The cleithrum is incompletely exposed, with the dorsal apex of the postbranchial lamina concealed by matrix and the overlying pelvic girdle (Fig 3C) while the anterior margin of the ventro-lateral lamina has been worn away. The visible area presents a broad, flat ventro-lateral section and a taller lateral section that tapers dorsally. The two sections meet at an angle of about 60 degrees at the level of a prominent pectoral spine (sp). The total length of the flattened ventrolateral margin (including the spine) is 25.5 mm. Ornament consists primarily of linear ridges with those on the spine being particularly elongate and thick. Sparse pores are present in the furrows between the ridges, particularly on the ventral surface. Ornamentation on the postbranchial lamina on the anterior half of the lateral section consists of closely-set denticulate tubercles, a feature also present in *Psarolepis* [27, 28] and placoderms [29].

The right dermal pelvic girdle (Figs 3B and 4A), as preserved in ventral view, is a sub-rectangular plate (12.9 mm along the lateral margin, including the spine) with ornament ranging from long linear ridges near the posterolateral margin to short ridges with interspersed pores over the remainder portion. The exposed lamina presents a gently curved anterior margin, a straight median margin and a slanted posterior margin that tapers posterolaterally towards a short cylindrical pelvic spine (pf.sp) as in *Psarolepis*, but differs from *Guiyu* which has a larger, more elongate spine [15]. A thickened lateral rim curves dorsally to meet a semi-exposed lateral lamina, and posteriorly forms a cylindrical strut resembling a slender version of the proximal portion of the pectoral fin spine.

Two elongate dermal elements lying adjacent to the displaced girdles are tentatively identified as an interclavicle (icl) and the left element of a pair of ventral scutes (Figs 1 and 3B). Alternatively, they may represent a median gular and left lateral gular, but this is unlikely owing to their proximity to the pectoral girdle and the absence of any other opercular-gular bones (submandibular or branchiostegal elements) in the preserved portion of the holotype. The interclavicle is a kite-shaped bone with a rostrocaudal length of 25.2 mm. The shorter anterolateral margins meet at a sharp anterior apex while the longer posterolateral edges, with slightly concave contour matching the adjoining ventral scute, taper gently towards a narrow, squared off posterior end.

#### Median dorsal and ventral plates

As in *Guiyu* [8,15], *Sparalepis* bears a single median row of large dorsal plates or ridge scutes, with large spines present on the plates immediately anterior to each of the two dorsal fins (Figs 1 and 3A). The dermal surface is reticulated with wavy ridges interspersed with numerous pores, contrasting sharply with the adjacent pore-less scales. A series of three plates (md1-md3) commences immediately behind the presumed location of the base of the skull. The anterior two are uniformly square shaped at about 8 mm in length, while the third is elongated (17.5 mm long at the base) and bears a large (30 mm long) dorsal spine (dfs1). A similar configuration exists in *Guiyu*, although the second plate of *Guiyu* has a concave posterior margin that embraces the spine-bearing third plate [15] whereas the margin in *Sparalepis* appears straight. As noted in Ref. 15, the original description of *Guiyu* erroneously combines the first and second plates due to uncertainty over natural margins versus artifacts of preservation [8, 15]. The presence of a first dorsal fin is inferred due to a posterior groove in the spine on the third plate followed by 17 mm gap in the median dorsal series, likely demarcating the basal extent of the fin web. A first dorsal fin was absent in the initial reconstruction of *Guiyu* [8], but is now believed to have been present due to similar reasoning [15].

Following the location of the presumed 1^st^ dorsal fin are a 4.5 mm long median dorsal scute (mds), only marginally larger than the adjacent scales, followed much larger 18 mm long plate (md4). Disruption of the median dorsal surface makes it unclear if additional plates were present leading up to the 2^nd^ dorsal fin spine (dfs2). The spine is broad-based and broken distally.

*Sparalepis* appears to have possessed paired ventral ridge scutes (vrs), as does *Guiyu* [15]. A lanceolate bone, 21.3 mm in length and posterolaterally adjacent to the interclavicle, is considered to represent the left example of the anterior-most pair of ventral scutes, with the corresponding margins of the interclavicle indicating the presence of a missing right scute. The convex medial margin of the scute bears a smooth overlap area for the median gular. Ornamentation comprises a reticulated arrangement of wavy ridges with numerous pores within the inter-ridge furrows.

A displaced rhombic plate lies adjacent to the second dorsal fin spine. It features pore-less linear ornament running perpendicular to the long axis. It is considerably larger (2.5mm length x 5.5mm height) than the flank scales in the immediate vicinity, and is similar in form and ornament to the paired ventral ridge scutes of *Guiyu* preserved immediately posterior of the pelvic girdle [15].

#### Scales

Recognizing and documenting the considerable variation in scale morphology on a single fish is essential for future identification of isolated scales from conspecific or related forms [30,31,32,33]. In describing the squamation of palaeoniscoid-grade actinopterygians, Esin [30] devised a scheme where the postcranial surface was divided into nine zones of distinct scale morphology. This method has been successfully employed by others in the description of Devonian actinopterygians [23,24,31] and other early osteichthyans with a rhombic scale morphology [27,33].

Although the flank scales do not display as prominent a reduction in dorso-ventral height along the antero-posterior flanks (Areas A to C) as in early actinopterygians [23,24,25,31], *Sparalepis* displays a broadly similar pattern of scale-variability with distinct morphology present within the areas designated by Esin [30]. As a result, Esin’s scheme will be used in this description (Fig 5A). Scales from the caudal fin and peduncle (Esin’s Area D) are not preserved. About 30 diagonal scale columns lie in front of the first dorsal fin base, as distinct from 15 comparable scale columns in *Guiyu* (condition in *Psarolepis* is unknown). All scales exposed in visceral view (completely visible in the mid-posterior flank and partially visible on the anterior flank) display a thickened base, separated from the crown via a distinct neck (Fig 6) as in *Psarolepis* [27], *Cheirolepis* [34] and some acanthodians [27], but not in *Guiyu*. Dermal ornamentation on the crown consists of parallel rostro-caudally directed enamel ridges, usually separate but with variable degrees of anastomosing on larger scales (Fig 5). There are no pores, unlike the ornament on the appendicular girdles and dorsal ridge scutes. As in *Psarolepis* [27], the scales lack a bony depressed field with the crown almost fully covering the base in crown view (Fig 5D).

Area A: Scales from the anterior flank (Figs 5B, 5C and 6C) are rectangular with a dorso-ventral depth of up to 5 times the antero-posterior length (up to 1.5 mm long x 7.5 mm tall). The anterior margins are straight whereas the posterior margins of the crown are serrated by the terminal portions of the ridges. Where sections of the squamation are missing, negative impressions of the scale base reveal a well-developed keel (k) flanked by prominent ledges (l.a, l.p). An anterodorsal bulge (p, p.ad), likely homologous with the combined peg and anterodorsal process of the "subtype 1" scales of *Psarolepis romeri* [27], slots into a shallow ventral socket (s) of the dorsally adjacent scale.

Each scale crown displays a prominent ventral bulge (vb) that slots into a pronounced concavity on the dorsal margin of the scale beneath it, effectively forming an interlocking dermal series of ventral ‘pegs’ and dorsal ‘sockets’. Scales partly out of articulation reveal a smooth surface (oa.vb) to accommodate the ventral bulge of the scale above it (Fig 5C) and reveal considerable overlap of the scale crowns. The anterior scales of *Sparalepis* thus possess separate dorso-ventral interlocking surfaces on the bases and the crowns (Fig 6C). The dermal articulations terminate at the 12^th^ scale row which is considered to be the transition zone with area B.

Area B: The scales on the mid-flank (Fig 5D) are of similar proportions to those of area A (up to 1.5mm long x 7mm tall) with the dorsoventral dimensions becoming gradually shorter down the length of the body. The crowns possess straight dorsal and ventral margins without the pronounced dorso-ventral interlocking surfaces of their anterior counterparts.

Area C: Scales from the posterior flank (aft of the approximate level of the 2^nd^ dorsal fin spine) are only preserved in basal view (Fig 6A and 6B). They are rectangular with a height of about 4 times the length (up to 1.2 mm long x 4.5mm tall). The base is thickened with a prominent keel wedged between the anterior and posterior ledges. The dorsal pegs and the articulating ventral sockets are broad, low and gently rounded. The anterodorsal process is large and club-shaped, underlying (in visceral view) the dorsal-most surface of the posterior ledge of the scale in front (Fig 6A and 6B).

Area E: Area E (Fig 5E) comprises the scales on the dorsal surface anterior of the 2^nd^ dorsal spine. At the transition zone with the flanks, the scales are rectangular with a height-length ratio of 3 to 3.5. There is a marked reduction in scale height with each ascending horizontal scale row. Scales adjacent to the median ridge scutes are rhombic with a roughly equal height to length ratio.

Area F: Scales on the lateral margins of the belly area are rectangular and drastically shorter (dorsoventrally) than the adjacent Area A-B scales with a dorso-ventral depth of twice the antero-posterior length. They lack the pronounced dorsal concavities of the adjacent Area A scales. Ventral to this, the scales are smaller and rhombic in shape, with a depth of about half the length.

Area G: Closely packed minute diamond-shaped scales, 1 mm in length and ornamented with one or two short ridges, are preserved immediately adjacent to the trailing edge of the 2^nd^ dorsal fin spine (Fig 6A). Additional scales in basal view are preserved in association with the Area C scales. The keel on these scales is considerably broader and more prominent than on the adjacent flank squamation while the peg and anterodorsal process are greatly reduced.

Area H: Scales from the posterior dorsal surface (Fig 6A) are preserved in basal view. They are similar to the Area G scales in being diamond shaped, lacking prominent peg or anterodorsal process and in having a greatly enlarged keel.

The presence of a distinctly palaeoniscoid-like arrangement of scale variability in non-actinopterygian osteichthyans like *Sparalepis* and *Andreolepis* [33] suggests that this type of squamation is plesiomorphic within crown-group Osteichthyes. *Guiyu* [8] displays a broadly similar configuration although the squamation of this taxon has yet to be described in detail.

## Discussion

### Phylogenetic results

The discovery of *Sparalepis* provides a second taxon of Silurian osteichthyan, along with *Guiyu*, known from a substantial portion of the skeleton (Fig 7). To explore the phylogenetic position of *Sparalepis*, we conducted phylogenetic analyses using the dataset presented by Qiao et al. [35] with the addition of character 336: “Relationship of crown and base of isolated trunk scale: crown fully covering the base (0); crown sitting on the bony base, with an exposed depressed field overlapped by adjacent scale in articulation (1)”. This dataset (S1 Text and S1 Dataset) was modified from that of Long et al. [36], which was in turn expanded and modified from previous analyses [8,37,38,39], and further expanded from Giles et al. [40], Brazeau and de Winter [41], and Lu et al. [42]. The character data entry and formatting were performed in Mesquite (version 2.5) [43]. All characters were treated as unordered and weighted equally, as in the earlier versions of this dataset.

Two agnathan taxa (Galeaspida and Osteostraci) were set as the outgroup. The dataset was subjected to the parsimony analysis in TNT software package [44]. The analyses were run using a traditional search strategy, with default settings apart from the following: 10,000 maximum trees in memory and 1,000 replications. Bremer support and bootstrap values were calculated using TNT [44], with heuristic searches.

Our analysis generated 2496 trees of 957 steps (CI = 0.3772; HI = 0.6228; RI = 0.8070; RCI = 0.3044), a strict consensus of which (Fig 8A) broadly agrees with the favoured hypothesis of Zhu et al. [12] with placoderms recovered as a paraphyletic array of stem gnathostomes, acanthodians as a paraphyletic array of stem chondrichthyans, and *Entelognathus* as the immediate sister group of crown gnathostomes. One most parsimonious tree (S1 Fig), which agrees well with the 50% majority-rule consensus (Fig 8B), is selected for illustrating inferred character transformations at various nodes.

*Sparalepis*, is consistently clustered with *Guiyu*, *Achoania* and *Psarolepis*; they together are positioned at the base of the Sarcopterygii, collectively forming the sister group of crown-group sarcopterygians. Previously described as a "*Psarolepis*-*Guiyu* cluster" [15], this putative clade is here informally referred to as the "psarolepids" for the sake of simplicity. Key synapomorphies uniting these taxa in this analysis (S1 Fig) include spines associated with the dorsal (character 122, state 1), pectoral (character 124, state 1), and pelvic fins (character 240, state 1); median dorsal plates (character 104, state 1); a dermal pelvic girdle (character 239, state 0) and an adsymphysial tooth whorl (character 41, state 1, their presence in *Guiyu* and *Psarolepis* is inferred on the basis of facets on the anterior dentaries of these taxa). These purported synapomorphies are problematic as all but the tooth whorls are present among the gnathostome stem-group (= placoderms). Additionally, adsymphysial tooth whorls are widely distributed among disparate branches of the gnathostome crown group (onychodonts, porolepiforms, the actinopterygian *Howqualepis* and the acanthodian *Poracanthodes*). In our analysis, large spines anterior to the appendicular and dorsal fins are lost in the stem-group Osteichthyes prior to the node containing *Dialipina* and crown-Osteichthyes. Median dorsal plates and plate-like dermal pelvic girdles are lost in the gnathostome stem prior to the node containing the osteicthyan and chondrichthyan total groups. Thus with the available data, the broad suite of placoderm-like characters present in the psarolepids are resolved as homoplasies.

At present the psarolepid cluster contains the totality of Silurian crown-gnathostomes known from reasonably complete remains. Both *Guiyu* and *Sparalepis* are known from articulated specimens whereas the disarticulated remains of *Psarolepis* are sufficiently comparable to these forms to reconstruct a substantial proportion of its anatomy [15,27,28]. The other two Silurian genera, *Lophosteus* and *Andreolepis*, are highly fragmentary as is *Meemannia* from the Lockhovian [45], recently posited as a stem-actinopterygian [46]. The Early Devonian *Dialipina* is known from complete specimens [47] that display an unusual (for an osteichthyan) dermal configuration of numerous small cranial and gnathal plates, presenting difficulties in determining homologies with other bony fishes [25] and possibly suggesting some degree of anatomical specialization. This taxon remains enigmatic and demands further scrutiny [12]. The placoderm-like characters highlighted in the psarolepids are also absent among the basal actinopterygians based on the available fossil record, although the clade that is not represented by reasonably complete material from before the Middle Devonian. While the presence of Silurian sarcopterygians necessitates the existence of Silurian actinopterygians, indisputable pre-Devonian representatives are currently unknown [48]. Given the striking anatomical disparity of forms like *Guiyu* and *Sparalepis* when compared to Middle-Late Devonian sarcopterygians, their discovery and description has profoundly enhanced our understanding of early sarcopterygian anatomy [8,15]. This may allude to the scope of our limitations regarding early actinopterygian evolution without the benefit of similarly complete Silurian fossil representatives.

An increasing body of evidence, including the strong morphological similarities of the paired-fin girdles and fin-spines between placoderms and psarolepids [8,15,28], similar dermal arrangements between the skulls of stem-gnathostomes *Entelognathus* [12, 49], *Qilinyu* [13] and *Janusiscus* [40] with crown-group Osteichthyes, and placoderm-like features noted in the early osteichthyan *Lophosteus* [50] provide compelling support for a placoderm-like ancestral bauplan for the osteichthyan total-group. When combined with our currently limited knowledge of pre-Devonian osteichthyan diversity and anatomy, it raises the possibility that the putative synapomorphies uniting the psarolepids in this analysis may ultimately prove to represent osteichthyan symplesiomorphies with the benefit additional fossil data. As such, it remains unclear at present as to whether the psarolepids constitute a genuine clade.

### The Xiaoxiang Fauna as an indicator of an early center of osteichthyan diversification in South China

Although the earliest record of putative gnathostomes may extend as far back as the Ordovician [51,52], until very recently the available fossil record of Silurian gnathostomes was scarce and highly fragmentary [1,2,3,10,16]. Prior to the discoveries of the exceptional Kuanti specimens [8,12,13,15], the only articulated fossil gnathostome dating from the Silurian was the acanthodian-like *Yealepis* from the Ludlow of Victoria, Australia, based on an incomplete postcranium [53]. Silurian ichthyofaunal assemblages in Eurasia and North America are dominated by 'ostracoderms', a paraphyletic grade of jawless fishes including heterostracans, thelodonts, galeaspids and osteostracans [16, 54] with gnathostomes generally being poorly represented, although there has been a substantial increase in fossil evidence in recent decades [54].

Recent discoveries from South China and Vietnam suggest a greater diversity of late Silurian jawed vertebrates than has previously been recognised [54]. For example, Silurian placoderm fossils are rare and putative in Eurasia and Gondwana but were apparently well established in the South China block based on well preserved and unambiguous remains, including antiarchs and arthrodire-like taxa [11,12,13,54].

With regards to bony fishes, the Ludlow Xiaoxiang Fauna displays a far greater diversity of osteichthyans than any other fossil assemblage of comparable age. Outside of the South China block, the pre-Devonian osteichthyan fossil record primarily comprises only two genera based on highly fragmentary remains. *Lophosteus*, with four described Silurian species and additional Devonian forms, is a widely distributed genus from the Baltic region, the Timan-Rechora region Arctic Canada and Australia, ranging in time from the Ludlow to Lochkovian [50,55,56]. *Andreolepis*, based on two species, is known from the Baltic region, northern Timan, the Central Urals and the Novavya Zemlya and Severnaya Zemlya archipelagos with a mid-Ludlow to Early Pridoli time range [33,52,56,57,58].

This contrasts sharply with the diversity of osteichthyans present in Ludlow Xiaoxiang Fauna with at least six taxa in the combined assemblage. Among these from the Kuanti Formation are the two only articulated Silurian taxa, *Guiyu oneiros* and *Sparalepis tingi*, as well as the largest pre-Devonian vertebrate, *Megamastax amblyodus* (Fig 9). At least two additional Kuanti forms are currently awaiting description based on recently prepared articulated specimens within the IVPP collections. Of the two scale-based Xiaoxiang taxa, *Naxilepis gracilis* is present in the Kuanti and the overlying Miaokao Formation while *Ligulalepis yunnanensis* is restricted to the Miaokao [10]. Pridoli sediments from South China are not as well sampled, but material from the Yulungssu Formation includes an indeterminable osteichthyan [5] and *Psarolepis romeri*, a species also present in roughly contemporaneous deposits in Vietnam [5,16,21].

The Devonian record of South China, combining the oldest fossil appearances of key groups and containing the most phylogenetically basal fossil taxa have established this region as a possible center of origin for several crown-group sarcopterygian lineages, including anatomically modern coelacanths [59], lungfishes [60] and tetrapodomorphs [61–63]. The discovery of diversified stem-sarcopterygians in the Silurian of South China reveals a rich regional history of osteichthyans extending as far back as the early Ludlow, indicative of an as yet largely unknown chapter in early gnathostome evolution well before the advent of the Devonian “Age of Fishes”.

## Materials and methods

### Field methods and preparation

The holotype (IVPP V17915) is permanently housed and accessible for examination in the collections of the Institute of Vertebrate Paleontology and Paleoanthropology (IVPP), Chinese Academy of Sciences, 142 Xizhimenwai Street, Beijing 100044, China. The fossil block was collected from the muddy limestone of the Kuanti Formation (Late Ludlow, Silurian) in Qujing, Yunnan, China and prepared mechanically by IVPP staff using pneumatic air scribes and needles under microscopes. No permits were required for the described study.

### Nomenclatural acts

The electronic edition of this article conforms to the requirements of the amended International Code of Zoological Nomenclature, and hence the new names contained herein are available under that Code from the electronic edition of this article. This published work and the nomenclatural acts it contains have been registered in ZooBank, the online registration system for the ICZN. The ZooBank LSIDs (Life Science Identifiers) can be resolved and the associated information viewed through any standard web browser by appending the LSID to the prefix "http://zoobank.org/". The LSID for this publication is: urn:lsid:zoobank.org:pub:B876FB8A-6A89-4547-9B49-3D9B27C8AF61. The electronic edition of this work was published in a journal with an ISSN, and has been archived and is available from the following digital repositories: PubMed Central, LOCKSS.

## Supporting information

S1 Text Extended results of phylogenetic analysis, consisting of: 1) taxa and principle sources of data; and 2) taxon-character matrix. (DOCX)

S1 Fig Extended results of phylogenetic analysis, presenting of one of 2496 most parsimonious trees to illustrate inferred character transformations at various nodes. (PDF)

S1 Dataset Nexus file containing the dataset of the phylogenetic analysis. (NEX)
