# Supplementary material for: A new osteichthyan from the late Silurian of Yunnan, China
Source: PLoS One. 2017 Mar 8;12(3):e0170929. doi: 10.1371/journal.pone.0170929 (PMC5342173; doi:10.1371/journal.pone.0170929)
Supplement: S1 Fig — (PDF) [file pone.0170929.s002.pdf]

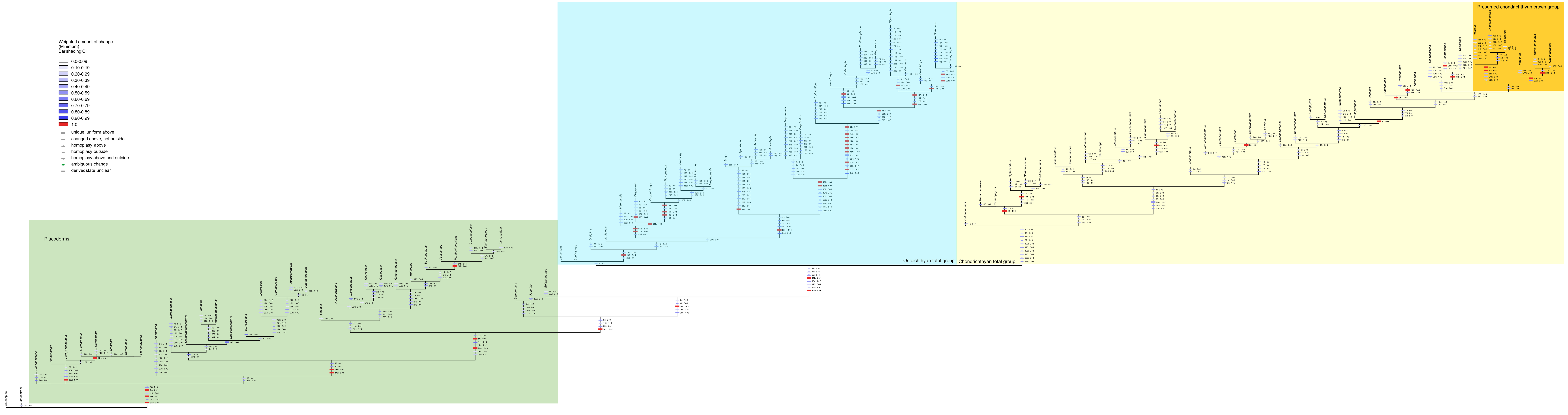

Weighted amount of change
(Minimum)
Bar shading:CI
0.0-0.09
0.10-0.19
0.20-0.29
0.30-0.39
0.40-0.49
0.50-0.59
0.60-0.69
0.70-0.79
0.80-0.89
0.90-0.99
1.0
unique, uniform above
changed above, not outside
homoplasy above
homoplasy outside
homoplasy above and outside
ambiguous change
derivedstate unclear
Placoderms
Osteichthyan total group
Chondrichthyan total group
Presumed chondrichthyan crown group
